# Reward processing and drug addiction: does sex matter?

**DOI:** 10.3389/fnins.2015.00329

**Published:** 2015-09-29

**Authors:** Liana Fattore

**Affiliations:** CNR Institute of Neuroscience-Cagliari, National Research Council-Italy, and Centre of Excellence “Neurobiology of Dependence,” Cittadella Universitaria di Monserrato, University of CagliariCagliari, Italy

**Keywords:** gender/sex difference, reward, addiction, sexual brain dimorphism, behavioral dependence

Reward processing is crucial to our health and wellbeing and dysfunctional brain reward signaling is a component of a number of psychiatric disorders, including major depression and drug addiction. Rewarding behaviors like eating, parenting, nursing, social play, and sexual activity are powerfully preserved in evolution and are essential for survival. All of them gratifying, they represent enjoyable experiences with high reward values and activate the same brain circuits that mediate the positive reinforcing effects of drugs of abuse. In line with preclinical findings and clinical observations, recent imaging studies confirmed that natural (sex, food) and non-natural (drugs of abuse) rewards differently activate male and female brains (Haase et al., 2011; Wetherill et al., 2014) and also that men and women differ in the ability to resist reward-related impulses (Diekhof et al., 2012). The study of sexual morphological differences in human brain has provided evidence of critical effects of gender on brain architecture and morphometry (Giedd et al., 2012; Feis et al., 2013). Male-female differences in human brain anatomy have stimulated research on the difference in onset, prevalence, and symptomatology of many neuropsychiatric illnesses between women and men, including drug addiction (Rando et al., 2013; Tanabe et al., 2013; Ide et al., 2014). Following the official recognition by International Institutions and Funding Research Agencies on the importance of taking into account potential differences between men and women in all of the relevant aspects of health-related research, gender is receiving increasing attention by medical and scientific communities. As a consequence, evaluation of *sex* and *gender* (i.e., biological characteristics and socio-political-cultural influences associated with the terms “male” and “female,” respectively) differences in reward processing in general, and in drug addiction in particular, is increasingly being studied.

Numerous human behaviors are driven by evolved instincts and urges. We have evolved the capacity to experience considerable pleasure and happiness from several behaviors, among which are eating, drinking, mating, creating protective shelter, and reproducing, all activating an anatomical and neurochemically defined brain circuit commonly referred to as the “pleasure circuit” or “brain reward system.” In both animals and humans, males and females display diverse attitudes and expectancies, process information differently, perceive experience and emotions in different ways and are behaviorally determined by different needs and drives. Reward processing may also differ between male and female population with sexual hormones playing an important, although not exclusive, role. When looking at the most common behavioral features known to favor the development of drug dependence, such as poor impulse control, risk-taking behavior, a heightened reactivity to stress and psychiatric comorbidity, all reveal important differences between men and women. Here I will illustrate the most recent evidence showing sex/gender differences in these and other behavioral traits linked to reward processing and enhancing the vulnerability to drug addiction.

## Sex differences in impulsivity, compulsivity, decision-making, risk-taking, and sensation-seeking

Impulsivity and compulsivity involve both repetitive behaviors and a deficit in delaying or inhibitory mechanism for such behaviors. Impulsivity is characterized by risky behaviors and attempts to maximize pleasure, arousal, or gratification, while compulsivity is characterized by hypervigilance, harm-avoidance, and ritualistic behaviors in the attempt to reduce anxiety or discomfort or to avoid potential threats. Converging evidence has long identified impulsivity and compulsivity as key psychological constructs in drug addiction (Perry and Carroll, 2008; Davison et al., 2014). A male-female unbalance has been recently observed in the prevalence and severity of disorders related to impaired impulse control capability (Liu et al., 2012; Perez-Rodriguez et al., 2012), including drug addiction (Mitchell and Potenza, 2015). Behavioral impulsivity consists of two distinct components: impulsive actions (also known as behavioral inhibition) that involve difficulty in inhibiting or controlling behavior, and impulsive choices that refer to the tendency to prefer smaller and immediate rather than larger and delayed rewards. Both of these components have been shown to play a role in several key transition phases of drug abuse (Perry and Carroll, 2008) and to differ among males and females (Weafer and de Wit, 2014). Notably, sex differences in the ability to control impulses have also been reported with respect to food intake (Galanti et al., 2007) and gambling (Tavares and Gentil, 2007).

Decision-making, which comprises a complex process of assessing and weighing short-term and long-term costs and benefits of competing actions, is another feature in which men and women differ (van den Bos et al., 2013; Sutterer et al., 2015). Risk-taking, defined as the tendency of choosing an action with high potential but unlikely profitable outcome over a less profitable/aversive alternative, is crucially linked to decision-making. Some studies have recently reported gender differences in risk attitudes (Zuckerman, 2006). For example, women display less risk-taking behaviors than men in various domains (Warshawsky-Livne et al., 2014) and score lower than men also on measures of sensation-seeking, i.e., the person's tendency to seek varied, novel, and intense sensations and experiences and to engage in novel or intense sensory activities (Cross et al., 2013). On the contrary, there is also evidence of no gender differences in risk attitudes (Lighthall et al., 2012; Derntl et al., 2014), thus highlighting the need for further research in the field.

Risk-taking and sensation-seeking are characteristic traits of adolescents which are known to give higher value to positive experiences (and lower value to the negative ones) than adults. Young people are more likely than adults to use illicit or dangerous substances, thus explaining why most drug addictions start during adolescence, with early drug use being associated with an increased rate of drug abuse and dependence. Adolescence has been unanimously recognized as the most critical phase in terms of vulnerability for addiction, during which behavior is strongly modulated by the sex hormones. Indeed, in addition to the perinatal period of sexual differentiation, adolescence is a sensitive period for steroid-dependent organization of the brain (and behavior) by steroid hormones, prompting a reassessment of the developmental time-frame within which organizational effects are possible (Schulz et al., 2009). Importantly, sex differences are now clearly emerging in drug use and abuse during adolescence (Kuhn, 2015).

## Sex differences in stress responsiveness and psychiatric comorbidity

It is quite obvious that stress alters the perception of rewarding stimuli and impacts on the pleasures that life may offer. Stress differentially affects male and female brain by engaging discrete regions involved in cognitive control, modulation of emotional processing, and neuroendocrine responses. Subjective stress, for example, is associated to stronger activations of the prefrontal cortex and deactivation of the orbitofrontal cortex in males, but to activations of limbic structures, like ventral striatum, insula and putamen in women (Wang et al., 2007). Stress favors the development and perpetuation of drug and alcohol use, but also the propensity to relapse in abstinent subjects (Sinha et al., 2006; Seo et al., 2011). Sex differences in stress responses have been observed in healthy and cocaine-dependent individuals (Kajantie and Phillips, 2006). Notably, in cocaine-dependent patients, cortico-striatal-limbic hyperactivity seems to be associated mostly to stress in women and to drug in men (Potenza et al., 2012), thereby suggesting a greater role of stress in triggering relapse in women than in men. However, it has also been reported that (i) men have greater hypothalamic-pituitary-adrenal (HPA) axis responses to a psychological stressor than women and (ii) women have greater hormonal reactivity than men to pharmacological stimulation with naloxone (Uhart et al., 2006). Imaging studies have allowed a better understanding of the link between stress and differential brain activation in either gender. For example, men display greater activation in brain regions known to regulate emotions during stress (Seo et al., 2011), in line with other studies describing greater emotional and physiological responses following stress in men compared to women, including enhanced cortisol levels (Kudielka and Kirschbaum, 2005), higher diastolic blood pressure responses (Chaplin et al., 2008), greater negative and aggressive emotions (Verona et al., 2007) and conditioned fear (Jackson et al., 2006). By contrast, during alcohol-cue exposures women showed greater neural activation in brain regions associated with high-level cognitive processing (Seo et al., 2011), consistent with the evidence of their tendency to express their emotions verbally and use verbal coping strategies more than men.

Closely related to stress, altered emotional states have also been reported to favor the propensity to drink alcohol and use drugs, and gender differences have been described in drug addiction and psychiatric comorbidities (Brady and Randall, 1999). Women suffer from anxiety and depression (Altemus et al., 2014) as well as from post-traumatic stress disorder (PTSD) (Peterlin et al., 2011) and eating disorders (Murnen and Smolak, 1997) more often than men. Negative affective states have been linked to drug abuse and addiction, thus it is not surprising that women drink alcohol mostly to ameliorate negative emotions (Abulseoud et al., 2013). In keeping with this, women use opioids more frequently than men to handle social pressure and anxiety (McHugh et al., 2013). Similar sex differences in negative affective states have been found in heavy drinking male and female adolescents (Bekman et al., 2013).

All these findings have clear potential clinical implications. Since stress plays a greater role in women than in men in initiating and maintaining drug use, treatments (either pharmacological or behavioral) targeted at reducing stress may have greater utility in patients with higher stress reactivity, i.e., in women. Indeed, behavioral therapies targeted at stress reduction, such as qigong meditation (which blends relaxation, breathing, guided imagery, inward attention, and mindfulness to elicit a tranquil state), seem to be more effective in women than in men in alleviating anxiety and withdrawal symptoms (Chen et al., 2010).

## Sex differences in drug use, craving, and relapse

Epidemiological studies consistently indicate that men typically use psychostimulants and alcohol more often than women. Men also smoke marijuana in greater amounts and at higher rates and more frequently use synthetic cannabinoids (UNODC, 2015). Men are also more likely to use illicit opiate drugs, but women more frequently abuse opioids through initial prescription painkiller use (Lee and Ho, 2013). Opiate-dependent women also report stronger cravings for opiates, and have higher “addiction severity index” and psychiatric severity scores than men (Back et al., 2011). Men have higher rates of cocaine use and abuse, but women with cocaine addiction tend to present a more severe clinical profile despite less drug use in terms of quantity and duration than their male counterparts (Greenfield et al., 2010). Male cigarette smokers are more likely to have a history of alcohol, cocaine, or marijuana abuse than female smokers which in turn suffer from psychiatric disorders (mostly anxiety or depression) more than men (Okoli et al., 2012). Accordingly, female smokers are more negatively affected by abstinence (Okoli et al., 2012; Pang and Leventhal, 2013) and experience stronger cravings than men (Perkins et al., 2013). Notably, this might explain why is more difficulty for women quitting in the longer term due to higher smoking-relapse and smoking-reinitiating rates (Ward et al., 1997). Sex differences in drug use, craving, and relapse can be ascribed, at least in part, to differences in the male and female brain morphology and function but also to gonadal hormones secreted early in development which masculinize and defeminize neural circuits, programming behavioral responses to hormones in adulthood (MacLusky and Naftolin, 1981).

## Sex differences in behavioral (not drug) addictions

Some behaviors may produce immediate reward, become persistent, and diminish control over it despite the knowledge of adverse consequences, thus leading to the so-called “behavioral addictions.” A behavioral addiction currently designates any behavior characterized by a sense of nervousness or awakening before committing the act followed by satisfaction and/or relief once committed it, other than the lack of ability to resist an urge or drive despite inevitably negative consequences. Thus, together with alcohol and substance abuse, behavioral addictions include compulsive eating, and sexual activity, pathological gambling, and Internet use, compulsive buying, and excessive exercising, kleptomania, and pyromania. All of these behaviors are often referred to as “impulse control disorders,” and are typically characterized by compulsiveness, impulsivity, impaired decision-making, craving, tolerance, withdrawal, and high rates of relapse. Notably, gender-related differences have also been described for these behavioral addictions (Fattore et al., 2014). Thus, men typically self-report higher score than women on gambling, sexual, or physical exercise activity, while women self-report higher score on compulsive shopping and food binging (Fattore et al., 2014). Studies based on small clinical samples indicate that the majority of treatment-seeking individuals with compulsive sexual behavior are males (Black et al., 1997). However, studies conducted on greater samples suggest less differences than previously thought (Reid et al., 2012), thus highlighting the importance of sample size and replicability when looking at gender differences.

## Future perspective

Awareness is increasing on the evidence that a different genetic makeup and hormonal milieu play crucial roles in sex-dependent differences in reward processing and drug addiction (Viveros et al., 2012). Sex-dependent differences have been described in many vulnerability factors that contribute to individual variation in the risk of drug addiction, including personality factors like impulsivity, sensation-seeking, stress responsiveness, and aversive states. Hormones as well as brain morphology and function have surely a part in the sex-dependent processing of rewarding stimuli, including drugs of abuse (Fattore et al., 2008). Recently, sexual dimorphism in drug-related neuroanatomic changes and brain-behavior relationships has been linked to the differences in clinical profiles and patterns of addiction between women and men (Regner et al., 2015).

A great progress has been made in the recognition and investigation of the differences between males and females in the field. Yet, well-controlled, adequately powered studies are necessary to gain a more complete picture of the degree to which males and females differ in drug and behavioral addictions. In this “translational era,” research is still in the need to face new challenges to deepen our knowledge on brain mechanisms through which male and the female brain processes rewarding stimuli (either natural or chemical) and lead to drug addiction, thus allowing the optimization of sex-tailored preventing strategies and treatment approaches for both genders.

### Conflict of interest statement

The author declares that the research was conducted in the absence of any commercial or financial relationships that could be construed as a potential conflict of interest.
